# Peutz-Jeghers Syndrome and lung cancer: does the risk meet the threshold for lung cancer screening?

**DOI:** 10.1007/s10689-026-00587-8

**Published:** 2026-07-03

**Authors:** Faizah Shareef, Mounica Konjeti, Samir Gupta, Jennifer M. Weiss

**Affiliations:** 1 Department of Medicine, University of California San Diego, San Diego, CA USA; 2 Department of Medicine, University of Vermont Larner College of Medicine, Burlington, VT USA; 3 Division of Gastroenterology & Hepatology, University of California San Diego, San Diego, CA USA; 4 School of Medicine and Public Health, Division of Gastroenterology & Hepatology, University of Wisconsin, Madison, WI USA

**Keywords:** Peutz-Jeghers Syndrome, Lung cancer screening, Cancer risk, Guidelines

## Abstract

Peutz-Jeghers Syndrome (PJS) is caused by germline pathogenic variants in the *STK11* gene and is associated with elevated lifetime risks for several cancers, including lung cancer. Currently, no formal recommendations exist for lung cancer screening in PJS. This structured narrative review compares lung cancer risk in PJS with lung cancer risk in individuals currently eligible for screening based on age and smoking history. PubMed and Web of Science were searched from 1/1/1980 to 9/11/2023 using the terms “Peutz-Jeghers Syndrome” AND “Cancer.” Studies reporting lung cancer risk in PJS were included. Reported lung cancer risks were compared to 5-year, 10-year, and lifetime lung cancer risks in the general population based on age and pack-years of smoking. Seventeen studies were included. Across the studies, the incidence of lung cancer ranged from 0.8 to 13.3%. Studies that included lung cancer risk relative to the general population reported a relative risk range of 2.9–22.9. Cumulative risk was reported in 5 studies and surpassed 5% by age 60 (range 7 to 17%). Guidelines for low-dose CT (LDCT) lung cancer screening for individuals aged 50–80 with a smoking history of at least 20 pack-years are associated with a similar or higher threshold for lung cancer risk. The lifetime lung cancer risk for individuals with PJS is similar to current guideline-based risk thresholds for screening based on age and smoking status. Given their elevated lifetime risk, individuals with PJS may be candidates for LDCT for lung cancer screening and should be formally studied to elucidate the risks, benefits, and optimal implementation in this patient population.

## Introduction

Peutz-Jeghers Syndrome (PJS) is a high-risk cancer predisposition syndrome attributable to pathogenic variants in the *STK11* gene, inherited in an autosomal dominant fashion. A clinical diagnosis of PJS can be established in a person who has one of the following characteristics: (1) two or more PJS-type hamartomatous polyps of the gastrointestinal tract; (2) any number of PJS-type polyps detected in a patient who has a family history of PJS in close relative(s); (3) mucocutaneous hyperpigmentation of the mouth, lips, nose, eyes, genitalia, or fingers in an individual who has a family history of PJS in close relative(s); (4) any number of PJS-type polyps in an individual who also has characteristic mucocutaneous pigmentation [1]. PJS confers increased lifetime risk for cancer in multiple organs including the small bowel, colon, breast, pancreas, ovaries, uterus, cervix, and lungs, with cumulative lifetime risk for 1 or more cancers as high as 93% without appropriate surveillance [2]. The National Comprehensive Cancer Network (NCCN) has well-established guidelines for small bowel and colon, pancreas, and breast cancer surveillance for patients with PJS [3]. NCCN guidelines also include recommendations for surveillance of the ovaries, cervix, and uterus. Although the NCCN guidelines note that PJS confers increased risk for lung cancer, they have no specific recommendations for or against lung cancer screening for patients with PJS, highlighting an important clinical practice gap for this population [3].

We postulate that screening for lung cancer in PJS could be considered if cumulative lung cancer risk is substantial, and if the cumulative risk exceeds thresholds which are used to justify screening for other risk paradigms, such as among middle-aged and older adults with longstanding history of tobacco smoking. A recent review of lung cancer risk among patients with PJS has not been conducted, and these risks have not been put in the context of current guideline-based risk thresholds that trigger lung cancer screening in the general population. This narrative review aimed to (1) identify reported lung cancer risk in PJS and (2) compare the observed risk to risks associated with other paradigms which are associated with recommendations for lung cancer screening.

## Methods

**Fig. 1:**
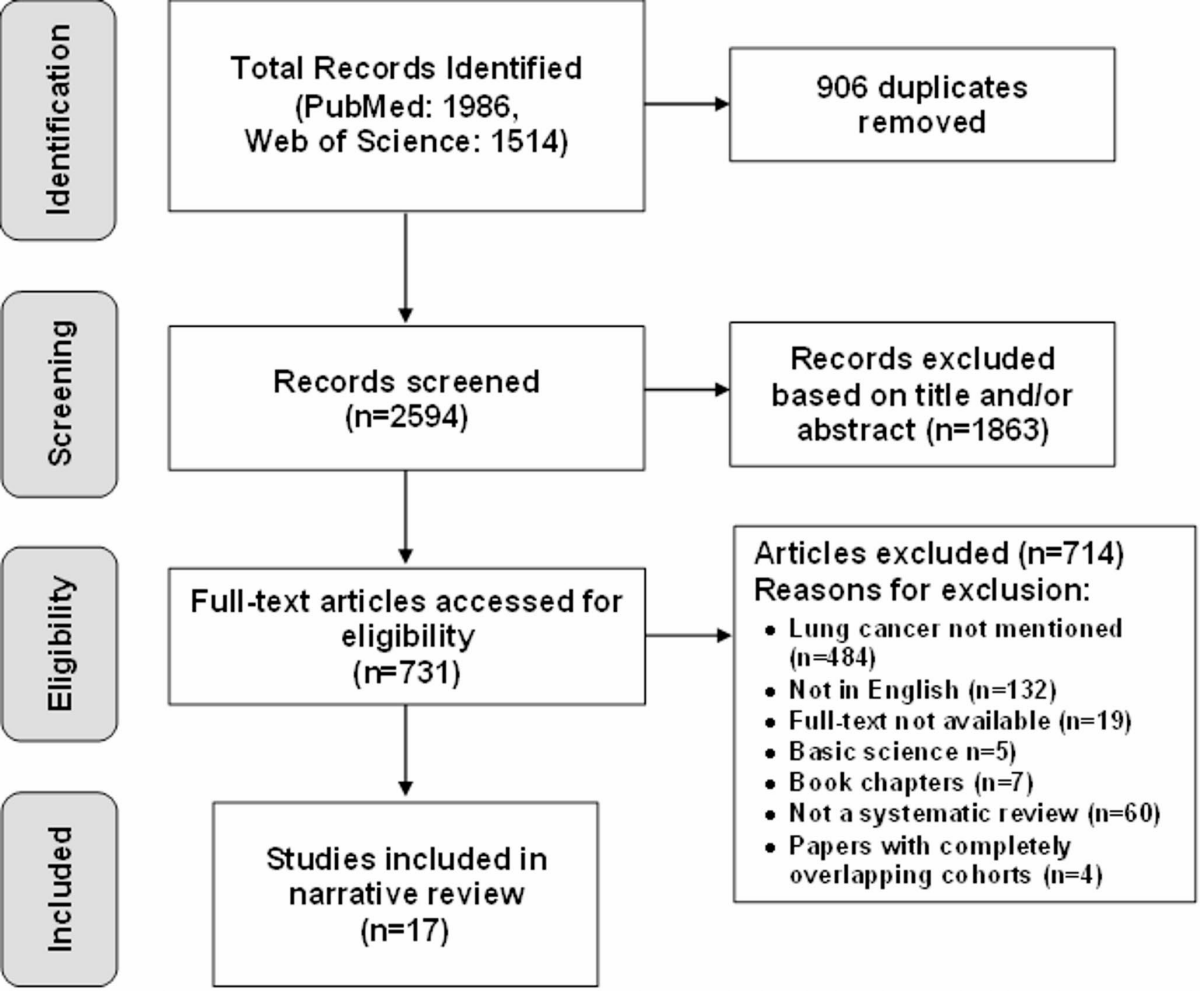
Flow diagram illustrating number of articles included/excluded at each stage of the screening process

We conducted a structured narrative review with the goal of identifying studies reporting on lung cancer risk in PJS, and guidelines for low dose computed tomography (LDCT) screening among those who smoke tobacco, including reported risk threshold for triggering screening. Both PubMed and Web of Science were searched from 1/1/1980 through 9/11/2023 using the terms “Peutz-Jeghers Syndrome” AND “Cancer” in all fields. We included cohort studies and case series/reports that reported on lung cancer risk in PJS (Fig. 1). Number of lung cancer cases, histology, and age at presentation were summarized, as well as method of lung cancer risk reporting. To better understand populations for which lung cancer screening was already recommended, current lung cancer screening guidelines from 15 groups were consolidated and separated out into screening group, pack-year thresholds for screening, and 5-year lung cancer risk data associated with populations for whom screening was recommended when available. A PubMed search was also performed to identify published articles elucidating lung cancer lifetime, 5-year, and 10-year risk for populations for which lung cancer screening is already recommended. Based on available information, we qualitatively assessed whether estimated lung cancer risk for individuals with PJS is similar, lower, or higher than risk among individuals for whom lung cancer screening is currently recommended based on pack-years of smoking and age.

## Results

### Lung cancer risk in PJS

Among the 3,500 articles screened, 17 unique studies specifically addressed lung cancer risk in PJS and were included for full text review (Table 1). The 17 articles consisted of 13 cohort studies and 4 case reports/case series. Adenocarcinoma was the most common lung cancer type in PJS patients, with five cases of bronchioloalveolar carcinoma and one case of large-cell undifferentiated lung cancer. Across the studies that reported proportion of PJS patients with lung cancer, the range was 0.8–13.3%. There was heterogeneity in the reported risks of lung cancer across the studies, with 2 studies calculating relative risks compared to the general country’s population and 5 studies calculating cumulative risks by age. The reported relative risks ranged from 2.9 to 22.9. Cumulative risk was reported in 2 of the 17 studies and surpassed 5% by age 60 (range 7 to 13%).

**Table 1:** Studies of lung cancer risk in PJS

Study	Country	Study Type	Sample	Histopathology Observed	Mean Age at Diagnosis (exact age if fewer than 3 cases)	Lung Cancer Risk
Giardiello et al. 1987 [4]	USA	Cohort study	31 patients with PJS from the Johns Hopkins Polyposis Registry	Adenocarcinoma of lung (*n* = 1) and Large-cell undifferentiated lung cancer (*n* = 1)	41 yrs (*n* = 1; large-cell undifferentiated lung cancer) 70 yrs (*n* = 1; adenocarcinoma)	Proportion with lung cancer was 6.5% (*n* = 2/31)
Spigelman et al. 1989 [5]	United Kingdom	Cohort study	72 patients with PJS from the St. Mark’s Polyposis Registry	Adenocarcinoma of lung assumed (specific histology not mentioned)	33 yrs (*n* = 1)	Proportion with lung cancer was 1.4% (*n* = 1/72)
Boardman et al. 1998* [6]	USA	Cohort study	34 patients with PJS from Mayo Clinic	Adenocarcinoma of lung assumed (specific histology not mentioned)	49 yrs (exact ages not reported)	Proportion with lung cancer was 8.8% (*n* = 3/34)
Hirano et al. 2002 [7]	Japan	Case report	31 yo Chinese male with sporadic PJS	Adenocarcinoma of lung	31 yrs (*n* = 1)	N/A
Lim et al. 2004 [8]	USA, Europe, and Australia	Cohort study	240 patients with PJS with an identifiable *STK11* mutation	Adenocarcinoma of lung assumed (ICD C34 = lung cancer NOS)	Not mentioned	Proportion with 1.2% (*n* = 3/240) Cumulative lung cancer risk by age: 1% (by age 40) 2% (by age 50) 7% (by age 60) 7% (by age 70)
von Herbay et al. 2005 [9]	Germany	Case report	22 yo male with PJS and an identifiable *STK11* mutation	Multilocular mucinous bronchioloalveolar carcinoma	22 yrs (*n* = 1)	N/A
Hearle et al. 2006 [10]	USA, Europe, and Australia	Cohort study	419 patients with PJS (Note: includes the 240 patients from Lim et al.)	Adenocarcinoma of lung assumed (ICD C34 = lung cancer NOS)	Not mentioned	Proportion with lung cancer was 1.9% (*n* = 8/419) Cumulative lung cancer risk by age: 1% (by age 40) 4% (by age 50) 13% (by age 60) 17% (by age 70)
You et al. 2010 [11]*	USA	Cohort study	54 patients with PJS from Mayo Clinic (likely partial overlap with the Boardman et al. 1998 paper)	Adenocarcinoma of lung assumed (specific histology not mentioned)	52 yrs (*n* = 1) 53 yrs (*n* = 1) 58 yrs (*n* = 1)	Proportion with lung cancer was 5.6% (*n* = 3/54)
van Lier et al. 2011 [12]	The Netherlands	Cohort study	133 patients with PJS	Adenocarcinoma of lung (*n* = 2) and bronchioloalveolar carcinoma (*n* = 2)	49 yrs (*n* = 1) (adenocarcinoma) 45 yrs (*n* = 1) (bronchioloalveolar carcinoma)	Proportion with lung cancer was 3.0% (*n* = 4/133)
Osoegawa et al. 2011 [13]	Japan	Case series	9 patients with lung cancer (2 PJS cases, 7 individuals without PJS)	Mucinous bronchioloalveolar carcinoma	43 yrs (*n* = 1) 68 yrs (*n* = 1)	N/A
Tchekmedyian et al. 2013 [14]	Uruguay	Cohort study	25 patients with PJS	Lung cancer (specific histology not mentioned)	50 yrs (*n* = 1)	Proportion with lung cancer was 4.0% (*n* = 1/25)
Chen et al. 2017 [15]	China	Cohort study	336 patients with PJS	Lung cancer (specific histology not mentioned)	Not mentioned	Proportion with lung cancer was 1.2% (4/336) RR of lung cancer was 22.91 (95% CI, 8.55–61.39)
Chiang and Chen 2018 [16]	Taiwan	Cohort study	15 patients with PJS	Lung cancer (specific histology not mentioned)	38 yrs (*n* = 1) 39 yrs (*n* = 1)	Proportion with lung cancer was 13.3% (*n* = 2/15)
Cortegoso Valdivia et al. 2020 [17]	Italy	Cohort study	24 patients with PJS	Lung cancer (specific histology not mentioned)	Not mentioned	Proportion with lung cancer was 8.3% (*n* = 2/24)
Wang et al. 2022 2022 [18]	China	Cohort study	412 patients with PJS	Lung cancer (specific histology not mentioned)	45 yrs (median age at diagnosis, range 24–69 yrs)	Proportion with lung cancer was 3.2% (*n* = 13/412) RR of lung cancer was 2.9 (95% CI, 1.5–4.9)
Fostira et al. 2022 [19]	Greece	Case report	34 yo male with PJS and an identifiable *STK11* pathogenic variant	Non-small cell adenocarcinoma of lung	34 yrs (*n* = 1)	N/A
Xu et al. 2023 [20]	China	Cohort study	566 patients with PJS identified based on ICD Q85.801; 361 patients had follow-up data	Lung cancer (specific histology not mentioned)	Not mentioned	Proportion with lung cancer was 0.8% (*n* = 3/361)

*These two studies covered overlapping time periods from the same institution – the 3 cases of lung cancer observed in each study may represent the same cases

The thirteen cohort studies included populations with PJS from the United States, Australia, Uruguay, China, Taiwan, The Netherlands, and Italy. Prior to 2010, there were five cohort studies [4–6, 8, 10] that included a total of 556 unique patients with PJS. These studies reported proportion of patients with lung cancer that ranged from 1.2% to 8.8%. In 2010, a new cohort study from the United States published by You et al. [11] included 54 patients with PJS from the Mayo Clinic registry from 1950 to 2002. Due to this time frame, there is likely partial overlap of patients from the Boardman et al. 1998 paper [6]. The median follow-up for individuals in this cohort was 7 years during which 3 individuals developed lung cancer (mean age at diagnosis 54 years). In 2011, van Lier et al. [12] published a cohort study of 133 patients with PJS from The Netherlands who were followed prospectively from 1995 to 2009 with 5,004 person-years of follow-up. This cohort included both familial and sporadic cases of PJS who were diagnosed by World Health Organization (WHO) diagnostic criteria [21], a proven *STK11* gene mutation, or both. Out of 133 individuals, 4 developed lung cancer (3%); two of these cancers were bronchioloalveolar carcinomas. Tchekmedyian et al. [14] reported on patients with PJS in the National Registry from Uruguay that were followed since December 2000. Their cohort consisted of 25 cases of PJS from 11 unrelated families. One individual in the cohort developed lung cancer (4%) at age 50 yrs. In a cohort of 15 Taiwanese patients with PJS [16], two patients developed lung cancer (13.3%) at a mean age of 38.5 yrs. The patients in this cohort were identified from pathology department and colorectal cancer registry data from a single hospital in Taiwan and were included if they fulfilled WHO diagnostic criteria for PJS. The mean follow-up interval from diagnosis of PJS to last follow-up or death in this study was 16.5 yrs (range, 5–28 yrs). Cortegoso Valdivia et al. [17] evaluated a retrospective cohort of 24 patients with PJS from Italy. It is important to note that the patients in this study comprised consecutive adult patients with a clinical or genetic diagnosis of PJS who underwent device-assisted enteroscopy or intra-operative enteroscopy from 2003 to 2019 at a tertiary care center. The focus of this study was evaluating the impact of an enteroscopy-based approach on reduction in gastrointestinal polyp burden, however, information on cancer diagnoses was also collected during the study timeframe where two patients were found to have lung cancer (8.3%).

Three of the cohort studies included are from China. Chen et al. [15] published a retrospective cohort study of 336 PJS patients of Han nationality from General Hospital of Air Force, People’s Liberation Army, enrolled from 2005 to 2016. In this cohort, four patients had lung cancer (1.2%). The authors calculated a relative risk of lung cancer in their PJS patients compared to the general Chinese population of 22.9 (95% CI, 8.5–61.4). In 2022, Wang et al. [18] reported on 412 Chinese PJS patients treated at Nanfang Hospital in Guangzhou who were followed prospectively from 2006 to 2021 contributing to 12,798 person-years of follow-up data. The patients were from 29 of 34 provinces in China. During the timeframe of the study, 13 individuals developed lung cancer (3.2%) with a median age at diagnosis of 45 yrs (range, 24–69 yrs). The reported relative risk of lung cancer in this sample compared to the general Chinese population was 2.9 (95% CI, 1.5–4.9). Xu et al. [20] followed 566 Chinese PJS patients from 1994 to 2022. At the final follow-up date of the study, 361 patients had been seen more than twice at their hospital. Out of these patients with sufficient follow-up data, three individuals had lung cancer (0.8%).

Four case reports/case series were also included in our review. Two of the case reports [7, 19] described men in their early 30s with adenocarcinoma of the lung. The patient in the Hirano et al. [7] case report was diagnosed with PJS based on clinical and histological criteria, but no genetic testing was performed. The patient in the Fostira et al. [19] case report had confirmatory genetic testing that identified a pathogenic *STK11* gene mutation. Both of these patients did not smoke tobacco. The other case report and case series included in this review [9, 13] describe patients with PJS and mucinous bronchioalveolar carcinoma who were ages 22 [9], 43 [13], and 68 [13] at diagnosis.

Taken together, available data suggest that patients with PJS do have increased risk for lung cancer, raising the question of whether surveillance could be beneficial.

### Limitations of data on lung cancer risk in PJS

While these studies shed light on the elevated risk of developing lung cancer in PJS patients and the crucial gap in caring for these patients given the current lack of formal lung cancer screening, there are limitations to consider. As with all rare diseases, observational studies are typically affected by ascertainment bias which can lead to overestimation of risks. The quality of the data in many studies is low due to relatively small sample sizes leading to wide confidence intervals. There are differences in years of follow-up, and variation of inclusion criteria applied for the PJS cohorts where some studies only included familial PJS and not sporadic PJS cases, while others included both. Moreover, studies reported lung cancer risk using various methods such as incidence rates, relative risk compared to the country’s general population, or cumulative risk by 10-year age intervals. Additionally, most studies did not include information on individuals’ smoking histories or additional confounding factors for the development of lung cancer, so it is difficult to disentangle risk due to lifestyle and environmental factors versus risk from PJS alone. Similarly, we cannot determine how lung cancer risk is impacted by age and smoking status in PJS based on current studies. Further, while multiple studies reporting increased lung cancer risk in PJS were found, no studies were identified that evaluated lung cancer screening, such as with chest x-ray or LDCT in patients with PJS.

### PJS risks in context of lung cancer risks among people who are eligible for LDCT screening due to tobacco use

Lifetime lung cancer risk among smokers ranges from 2.5 to 13.4% [22], while 10-year risk estimates are 5–11% for males and 4–6% for females who smoke [23, 24]. In contrast, the 10-year risk among non-smokers is only 0.4–0.8%. Lung cancer risk generally increases with age, peaking between 60 and 70 years before declining.

Lung cancer screening guidelines from multiple countries/regions are summarized in Table 2. Many recommend annual or biennial low-dose computed tomography (LDCT) for high-risk individuals. The U.S. Preventive Services Task Force (USPSTF) advises screening for adults aged 50–80 years with a ≥ 20 pack-year smoking history who currently smoke or quit within the past 15 years. The 5-year risk of developing lung cancer for those meeting USPSTF criteria is 3.9%, with a 2.5% risk of death [25]. USPSTF criteria are supported by the American Cancer Society, American Academy of Family Physicians, and American Lung Association [26, 27]. The American Association for Thoracic Surgery (AATS) recommends screening individuals aged 55–79 years with ≥ 30 pack-years or individuals aged ≥ 50 years with ≥ 20 pack-years and additional risk factors (e.g., COPD, genetic susceptibility, prior cancer) if their 5-year lung cancer risk exceeds 5% [28, 29]. The American College of Chest Physicians (ACCP) offers a conditional recommendation for annual screening when the 5-year lung cancer mortality risk exceeds 1.33% [30].

Non-US lung cancer screening recommendations are more likely to include risk model-based selection rather than relying solely on age and smoking pack-year cutoffs. The European Respiratory Society notes that multiple prediction models provide a more efficient method to identify individuals eligible to participate in lung cancer screening and have yielded higher detection rates in multiple trials and pilot programs [31]. Programs in England, Canada (Ontario), and Germany have demonstrated that risk models identify more lung cancers per screen compared to simple age and pack-year cutoffs [32–34]. Recommendations from some Asian countries include risk factors for non-smokers such as family history, exposure to second-hand smoke, and occupational exposures [35, 36]. Japan is unique among high-income nations in continuing to recommend annual chest x-ray as the predominant screening modality and saving LDCT for high-risk criteria [36].

**Table 2:** Summary of Current Lung Cancer Screening Guidelines for the General Population

Organization/National Cancer Screening Program	Year	Screening group	Lung cancer risk threshold (pack years/total cumulative exposure)	Screening and risk management recommendations	Lung cancer risk threshold (cumulative risk over time)
US: U.S. Preventive Services Task Force (USPSTF) [37]	2021	Ages 50–80 years	20 pack-years and currently smoke or have quit within the past 15 years	Annual screening with low dose computed tomography (LDCT) scan for the indicated risk group. Stop screening once a person has not smoked for 15 years or has a health problem that limits life expectancy or the ability to have lung surgery.	5-year risk > 3.9% or death risk > 2.5%
US: American Association for Thoracic Surgery (AATS) [29]	2012	Group 1: Ages 55–79 years Group 2: Age ≥ 50 years	Group 1: 30 pack-years Group 2: 20 pack-years if there is an additional cumulative risk *≥* 5% over the following 5 years.	Annual screening with LDCT scan for ages 55–79 with ≥ 30 pack-year smoking history. Annual screening with LDCT scan for ages ≥ 50 years and ≥ 20 pack-year smoking history and one additional risk factor (COPD with FEV1 < 70%, environmental/occupation exposure, prior cancer/radiation therapy, genetic/family history).	Cumulative 5-year risk greater than or equal to 5%
US: American Cancer Society (ACS) [38]	2023	Ages 50–80 years	≥ 20 pack-years and currently smoke or previously smoked	Annual screening with LDCT scan for ages 50–80 years with ≥ 20 pack-year smoking history, either currently smoking, or have previously smoked, and who are in relatively good health	N/A
US: American College of Chest Physicians (CHEST) [30]	2021	Ages 55–77 years	30 pack-years, continued smoking or have quit within 15 years	Annual screening with LDCT scan for ages 55–77 years with ≥ 30 pack-year smoking history and either continue to smoke or have quit within the past 15 years.	5-year incidence risk ≥ 2% on Lung Cancer Risk Assessment Tool Calculator or 6-year risk ≥ 2.6% on the Prostate, Lung, Colorectal, and Ovarian calculator or 10-year risk ≥ 5.2% on the Bach calculator and ≥ 10 years of life expectancy
US: National Comprehensive Cancer Network (NCCN) [39]	2026	Age ≥ 50 years	≥ 20 pack-year history of smoking cigarettes or ≥ 20-year history of smoking cigarettes	Annual screening LDCT scan for individuals ≥ 50 years with ≥ 20 pack-year history of smoking cigarettes or ≥ 20-year history of smoking cigarettes	6-year lung cancer risk threshold of about 1.3%
European Society of Thoracic Imaging (ESTI) [40]	2026	Ages 50–75 years	≥ 20 pack-year history of smoking (includes both current and ex-smokers)	Annual screening with LDCT (or biennial after a negative scan) for the indicated risk group. Scans must be read by trained and experienced radiologists and incorporate deep learning technology to assist with nodule detection	N/A
Australia: National Lung Cancer Screening Program [41, 42]	2025	Ages 50–70 years who are asymptomatic	≥ 30 pack-years, currently smoking or have quit within 10 years	Biennial screening with LDCT for the indicated risk group. Follows Pan Can nodule management protocol for baseline scan [43].	Prostate, Lung, Colorectal, and Ovarian 2012 model (PLCOm2012) [44] 6-year lung cancer risk score ≥ 1.51%
Canada: British Columbia Lung Screen Program [45]	2026	Ages 55–74 years	Current or former smoker with ≥ 20 years of smoking history	Biennial screening with LDCT for the indicated risk group	PLCOm2012 6-year lung cancer risk score ≥ 1.51%
Canada: Ontario Lung Screening Program [46]	2025	Ages 55–80 years	Current or former smokers who have smoked cigarettes daily for at least 20 years (not necessarily 20 years in a row)	Annual screening with LDCT for the indicated risk group	PLCOm2012 6-year lung cancer risk score ≥ 2.0%
England: National Health Services Lung Cancer Screening Programme [47]	2025	Ages 55–74 years	Ever smokers with risk threshold based on PLCOm2012 and LLPver2 models	Biennial screening with LDCT for the indicated risk group	PLCOm2012 6-year lung cancer risk score ≥ 1.51%; Liverpool Lung Project version 2 (LLPv2) [48] 5-year risk score ≥ 2.5%
Taiwan: National Lung Cancer Early Detection Program [35]	2022	Ages 50–74 years, unless eligibility is due to family history risk factor (males: 50–74 years; females: 45–74 years)	Smokers: ≥30 pack-years, are willing to quit smoking or have quit within the past 15 years Non-smokers: family history of lung cancer in parents, siblings, or children	Biennial screening with LDCT for the indicated risk group	N/A
Japan: Ministry of Health, Labour, and Welfare [49]	2018	Age ≥ 40 years (both smokers and non-smokers)	Smokers: Age 40 and older for general screening (chest x-ray and sputum cytology); LDCT for higher-risk individuals	Smokers: Annual chest x-ray and sputum cytology with LDCT as an opportunistic screening method for individuals 50 and older with Brinkman index ≥ 600 (e.g., ≥ 30 pack years of smoking) Non-smokers: Annual chest x-ray	N/A
Germany: Federal Joint Committee decision [50]	2025	Ages 50–75 years and participation in the statutory health insurance system	Individuals with at least a 25-year history of smoking and have accumulated at least 15 pack-years of cigarette smoking who are current smokers or have quit with the past 10 years	Annual LDCT for the indicated risk group	PLCOm2012 6-year lung cancer risk score ≥ 1.58% (supported by the HANSE study) [34]
South Korea: National Lung Cancer Screening Program (NLCSP) [51]	2019	Ages 54–74 years	≥ 30 pack-years, currently smoking or have quit within 15 years	Biennial LDCT for the indicated risk group. Utilize a modified, localized version of the Lung Imaging Reporting and Data System (Lung-RADS) optimized to reduce false positives caused by the high prevalence of tuberculosis in the region.	N/A
China: National Guidelines for the Screening and Early Detection of Lung Cancer [36]	2021	Ages 50–74 years and high-risk	High-risk criteria include at least one of the following: - ≥ 30 pack-years, including former smokers who quit within the last 15 years - living or working in the same room with a smoker for ≥ 20 years - at least 1 year of occupational exposure to carcinogens (e.g., asbestos, beryllium, uranium, or radon) - COPD or diffuse pulmonary fibrosis - family history of lung cancer in a first-degree relative	Annual LDCT for the indicated risk group	N/A

## Discussion

The premise of this study was to quantify lung cancer risk in PJS patients and determine how it compared to lung cancer risk in patients for whom lung cancer screening is recommended in the general population based on age and smoking history. This review addresses a gap in current clinical screening guidelines and practices for patients with PJS. Our results show that the incidence of lung cancer in the PJS patients included in the cohort studies was 0.8–13.3%. The studies that included relative risk assessments for lung cancer reported a range of 2.9–22.9 compared to that country’s general population. Cumulative risk was reported in 5 studies, and in all of those reports, surpassed 5% by age 60.

Existing literature includes two systematic reviews/meta-analysis that were published in 2000 [2] and 2010 [52]. The meta-analysis by Giardiello et al. [2] included 6 studies with a total of 210 patients. The relative risk for all cancers was 15.2 (95% CI, 2–19), and the cumulative risk for all cancers from ages 15–64 was 93% in individuals with PJS. A total of 5 cases of lung cancer were observed in the 210 patients with a statistically significant increase in relative risk for lung cancer (RR = 17, 95% CI, 5.4–39) and the cumulative risk of lung cancer from ages 15–64 yrs reported as 15%. Not all of the studies included in the Giardiello et al. meta-analysis addressed lung cancer risks. The systematic review by van Lier et al. [52] included a total of 21 articles (20 cohort studies, 1 meta-analysis) up through February 2009. There was overlap in the patient population included in the studies in this narrative review and definitions for patient inclusion were varied. Ultimately, a total of 1,644 patients with PJS were included in the 20 cohort studies. Among these patients, there were 25 cases of lung cancer with a mean age of 47 years at diagnosis. Based on information from three studies included in the van Lier systematic review [2, 8, 10], the cumulative risk of lung cancer by age 60–65 years was reported as 7–17%. Our narrative review adds significantly to these prior studies by including 12 additional cohort studies and case reports/case series that specifically address lung cancer risk in PJS patients and comparing the outcomes to lung cancer risks that trigger recommendations for lung cancer screening in the general population. Based on our review, the cumulative risk for lung cancer in PJS is similar to the risk of lung cancer in people who smoke and meet criteria for lung cancer screening by organizations such as the USPSTF, American Association for Thoracic Surgery, and the NCCN. This supports the notion that patients with PJS may be considered for lung cancer screening with annual LDCT.

Lung cancer screening using LDCT has demonstrated benefits, including a 20% reduction in lung cancer mortality among high-risk individuals, as evidenced by the National Lung Screening Trial [53, 54], which also highlighted the importance of early detection for improved survival rates. The absolute risk reduction was about 3 per 1,000 screened, meaning 3 fewer lung cancer deaths for every 1,000 people screened. Based on this data, LDCT lung cancer screening has been adopted in the US, however, European countries have been more cautious about widespread implementation of these programs. Early trials in Europe had significant heterogeneity in cohort selection criteria, screening interventions in the control group, frequency of screening, definitions of positive screening, and nodal management strategies making it difficult to directly compare the results [55]. Due to this heterogeneity, there is currently no global consensus on how to implement these programs, including how to determine eligibility for participation, optimal intensity of screening, or optimal duration of screening. Other considerations include risks associated with screening such as a high false-positive rate of about 23.3%, potential overdiagnosis, unnecessary procedures, and exposure to low doses of radiation. With all of this in mind, benefits of lung cancer screening were observed in some studies in individuals with either a 5-year lung cancer risk greater than 3.9% or a cumulative risk of at least 5% [56, 57]. Since the studies that reported a cumulative risk of lung cancer in PJS patients showed a risk exceeding 5% by age 60, it is reasonable to suggest that they could also benefit from lung cancer screening. Such recommendations would need to be individualized, taking into account absent data on impact of screening specific to PJS, patient preferences, and potential for false positive results requiring further work up [58, 59]. Further, while radiation-associated breast cancer risks related to LDCT have been reported to be low, women with PJS at baseline are at markedly higher lifetime risk for breast cancer, and decision making regarding whether to offer LDCT for lung cancer screening will need to be individualized [59, 60]. At minimum, clinicians caring for individuals with PJS should vigilantly monitor for early signs and symptoms of lung cancer.

Several strengths and limitations may be considered in interpreting our analysis. The study directly addresses a gap in NCCN guidelines for PJS patients, which recognize increased risk for lung cancer but do not offer recommendations for surveillance. Our findings may allow clinicians to engage in shared decision making with PJS patients that can help them decide if they would like to pursue lung cancer screening. We anticipate that these discussions may need to consider factors such as radiation exposure (particularly for women who have not elected for risk reducing mastectomy), and the potential for identification and management of incidental findings. We do note that our estimates of lung cancer risk among PJS patients were not able to capture the influence of other factors on lung cancer risk, such as smoking status or environmental exposures. Additionally, the data for PJS from case reports (especially older case reports) may not reflect the current risks and demographics of PJS populations. An updated analysis of lung cancer risk in individuals with known *STK11* pathogenic variants, utilizing resources such as the UK Biobank [61], the Department of Veterans Affairs Million Veteran Program [62], or the National Institutes of Health-sponsored All of Us Study [63] could provide more modern estimates of lung cancer risk for individuals with PJS.

In summary, those with PJS have an estimated lifetime lung cancer risk that exceeds current guideline thresholds for lung cancer screening based on age and smoking status.

Direct evidence supporting lung cancer screening in PJS is lacking. However, when contextualized against risk thresholds used for screening those who smoke tobacco, the elevated lifetime lung cancer risk in PJS suggests LDCT screening could be considered for this population and should be formally studied to elucidate the risks, benefits, and optimal implementation. At minimum, clinicians caring for those with PJS should remain vigilant in monitoring for signs and symptoms of lung cancer, and counseling regarding harm reduction strategies, such as avoiding tobacco smoking.

## Data Availability

No datasets were generated or analysed during the current study.
